# Xenobiotic Receptors and Their Mates in Atopic Dermatitis

**DOI:** 10.3390/ijms20174234

**Published:** 2019-08-29

**Authors:** Deborah Minzaghi, Petra Pavel, Sandrine Dubrac

**Affiliations:** Department of Dermatology, Venereology and Allergology, Medical University of Innsbruck, 6020 Innsbruck, Austria

**Keywords:** atopic dermatitis, xenobiotic receptors, pollution, PXR, AHR, PPAR, LXR, skin, inflammation

## Abstract

Atopic dermatitis (AD) is the most common inflammatory skin disease worldwide. It is a chronic, relapsing and pruritic skin disorder which results from epidermal barrier abnormalities and immune dysregulation, both modulated by environmental factors. AD is strongly associated with asthma and allergic rhinitis in the so-called ‘atopic march’. Xenobiotic receptors and their mates are ligand-activated transcription factors expressed in the skin where they control cellular detoxification pathways. Moreover, they regulate the expression of genes in pathways involved in AD in epithelial cells and immune cells. Activation or overexpression of xenobiotic receptors in the skin can be deleterious or beneficial, depending on context, ligand and activation duration. Moreover, their impact on skin might be amplified by crosstalk among xenobiotic receptors and their mates. Because they are activated by a broad range of endogenous molecules, drugs and pollutants owing to their promiscuous ligand affinity, they have recently crystalized the attention of researchers, including in dermatology and especially in the AD field. This review examines the putative roles of these receptors in AD by critically evaluating the conditions under which the proteins and their ligands have been studied. This information should provide new insights into AD pathogenesis and ways to develop new therapeutic interventions.

## 1. Atopic Dermatitis

Atopic dermatitis (AD) is the most common inflammatory skin disease, predominantly affecting young children and characterized by relapsing pruritic eczematous lesions over dry skin. AD affects 1%–37% of children and 1%–20% of adults worldwide and became a major health problem among children born after 1980 (http://isaac.auckland.ac.nz/index.html). Importantly, AD is considered the initial step of the so-called ”atopic march”. Indeed, while 70% of children afflicted with AD experience a full remission at 10–12 years of age, as many as 20%–70% of them go on to develop asthma, allergic rhinitis and/or food allergies [1]. Although significant etiological and therapeutic progress has been made in the past 30 years, the problem of AD continues to spiral out of control. 

AD is a chronic relapsing skin disease whose pathogenesis is not yet fully understood. However, there is consensus that epidermal barrier impairment precedes the development of immune hyper-responsiveness in both AD [2,3,4] and allergies [5]. Impaired epidermal barrier function likely results from a combination of environmental, genetic and epigenetic factors, and has been best studied in the context of loss-of-function mutations in the filaggrin gene (*FLG*) [6,7]. *FLG* is located on chromosome 1 in a region known as the epidermal differentiation complex, which contains genes encoding epidermal structural proteins and whose genetic variants have been repeatedly associated with AD [8]. However, other genetic variants have also been identified but with a weaker association strength [9]. The environmental factors involved in AD, recently designated as the exposome, are mainly stress, food, pollution and the skin microbiota [10]. The skin microbiota is altered in AD, beginning with dysbiosis in non-lesional AD and culminating with *Staphylococcus*-mediated superinfection in lesional AD. AD patients exhibit reduced diversity of the bacterial skin community because of enrichment in *Staphylococci*—*Staphylococcus aureus* in the severe cases, *Staphylococcus epidermidis* in the milder forms of AD—which is suspected to contribute to disease pathogenesis and flares [11,12,13,14]. Moreover, previous work has shown a trend of increased-and-fungal diversities at the genus and species levels, with higher frequencies of *Malassezia sympodialis*, *sloofiae* or *dermatis*, in AD, depending on sampling methods [15,16,17]. Approximately 80% of AD patients show IgE-mediated sensitization and positive skin prick tests for *Malassezia* [18]. *Malassezia* may actively contribute to the development of AD by degrading host skin lipids, thereby exacerbating initial skin barrier impairment. The role and composition of the skin microbiota in AD have been reviewed recently [19,20,21]. Thus, environmental factors synergize with (epi) genetic predisposition to weaken the epidermal barrier, and thus trigger AD. 

Primary epidermal barrier defects provoke compensatory responses, such as keratinocyte (KC) hyper-proliferation, leading to epidermal thickening and hyperkeratosis [22]. Consistent with this, increased numbers of Ki67^+^ KCs and up-regulation of keratin (KRT) 16 have been found in AD skin. Furthermore, various cytokines (IL-1, TNF-α) and growth factors (GM-CSF) are secreted by epidermal cells in order to sustain KC proliferation and metabolic requirements, such as DNA and lipid synthesis. Abnormal lipid metabolism has also been uncovered in AD skin, but it remains unclear whether it results from genetic variants affecting lipid-related genes, the epidermal differentiation of complex genes or from non-genetic events. Moreover, unfortunately, the literature is contradictory on skin lipid-content in AD, precluding a clear view of associated lipid changes [23,24,25,26,27,28,29]. However, there is agreement on a shift from very long chain to shorter-chain fatty acids (FAs) in ceramides of the stratum corneum in AD skin, regardless of skin lesions and *FLG* null mutations, thus causing further disruption of the lamellar bilayer organization [27,29,30,31,32]. Moreover, the amounts of ω3 and ω6 polyunsaturated FAs (PUFAs) and of their downstream metabolites are altered in AD skin [25,33,34]. These results are in line with transcriptomic analyses showing dysregulated expression of lipid-related genes in AD skin [35,36,37].

The current hypothesis on the primary role of epidermal barrier impairment in initiating the immune abnormalities in AD includes the secretion of alarmins (IL-25, HMGB1, IL-33, IL-1 and TSLP) by damaged KCs [38], which in turn prime Langerhans cells (LCs) to initiate a local Th2 immune response [39,40,41]. However, the immune abnormalities accompanying the epidermal barrier impairment in AD are complex. AD skin is abundantly infiltrated with various immune cells, including T-lymphocytes, inflammatory dendritic cells, mast cells, eosinophils and innate lymphoid cells-2 [22,42,43]. AD is considered a Th2-driven inflammatory skin disease in which a Th2/Th17 or Th1/Th17/Th22/Th9/Th2 immune response is observed depending on the disease status (nonlesional versus lesional, respectively). Immune abnormalities in AD have been extensively reviewed recently [44,45].

## 2. Xenobiotic Receptors and Mates

Xenobiotics are defined as molecules found within an organism, which are not naturally produced by or expected to be present in this organism. Thus, the term xenobiotic includes environmental pollutants, carcinogens, drugs, food additives and pesticides, as well as microbial-derived metabolites. Xenobiotic receptors regulate the detoxification processes of exogenous (e.g., pollutants and drugs) and endogenous (e.g., bile acids and bilirubin) compounds whose accumulation in cells induces cellular damage. Such molecules bind to and activate the receptors, whose signaling results in upregulation of the expression of a panel of detoxification enzymes and membrane transporters involved in metabolite uptake and efflux. In turn, these enzymes and transporters promote the elimination of the toxic compounds. However, the role of xenobiotic receptors encompasses more than xenobiotic metabolism. Accumulating evidence has implicated their involvement in many cellular processes, including energy homeostasis, cell proliferation, inflammation, tissue injury and repair, the immune response and cancer development [46]. 

The pregnane X receptor (PXR, NR1I2), constitutive androstane receptor (CAR, NR1I3), aryl hydrocarbon receptor (AHR) and peroxisome proliferator-activated receptors (PPARs) are stricto sensu xenobiotic receptors [47]. Liver X receptors (LXRs) and farnesoid X receptor (FXR, NR1H4) are related receptors that engage in crosstalk with xenobiotic receptors through, for example, protein–protein interactions between common co-activators. Indeed, competition between such co-factors for binding to receptors is a key mechanism for the regulation of physiological processes [48]. Moreover, some receptors can control the expression of others or be activated by the same ligands [49,50,51]. Xenobiotic receptors are all promiscuous receptors able to be activated by a broad range of molecules and involved in the transcriptional regulation of genes encoding Phase I (e.g., CYP450s) and Phase II enzymes (e.g., UGTs, SULTs and GSTs), as well as uptake and efflux transporters (e.g., MRPs and MDRs). Xenobiotic receptors and their mates are all highly expressed in the liver and intestine, but they have recently captured the attention of skin researchers because of their potential role in the skin and especially in AD pathogenesis. Indeed, they are expressed in skin epithelial and immune cells and dermal fibroblasts [52,53,54,55,56] where they control the expression of a panel of key genes belonging to pathways implicated in AD pathogenesis; i.e., lipid metabolism, cell proliferation and death, oxidative stress and immune response [55,57,58,59,60,61,62,63,64]. Moreover, their potential ligands are produced by skin microbes or present in the environment. This review will focus on receptors that are relevant in dermatology and especially in AD; i.e., AHR, PXR, LXRs and PPARs. The role of PPARs and LXRs in the skin has already be extensively reviewed [65,66,67,68], whereas the role of CAR and FXR in the skin and in AD has not yet been investigated. 

### 2.1. AHR

AHR is a ligand-activated transcription factor of the basic, helix-loop-helix motif-containing Per-ARNT-Sim family [69]. AHR is activated by a large number of halogenated aromatic hydrocarbons, including dioxins such as TCDD, polychlorinated biphenyls (PCBs) and polycyclic aromatic hydrocarbons (PAHs) (e.g., BaP, 3-methylcholanthrene); clinically used drugs (e.g., omeprazole); food-derived molecules, such as flavonoids (e.g., quercetin and resveratrol); and endobiotics (e.g., bilirubin, FICZ and metabolites of arachidonic acid) [55,70,71,72,73]. AHR ligands can also be secreted by bacteria [55]. AHR regulates the transcription of genes encoding phase I (i.e., *CYP1A1*, *CYP1A2*, and *CYP1B1*) and phase II (i.e., *UGT1A1*, *UGT1A3* and *UGT1A4*) enzymes and of efflux transporters (i.e., *ABCG2*) [72,74,75,76,77,78]. In the absence of ligands, AHR resides in the cytoplasm where it forms a protein complex that includes Hsp90, XAP-2 and p23 [79,80]. After ligand binding, AHR dissociates from this complex and translocates to the cell nucleus to bind to DNA responsive elements, also referred to as xenobiotic responsive elements, after interaction with nuclear-localized co-activators or co-repressors [73]. The AHR repressor protein, AHRR, enables the binding of corepressors to AHR and promotes its degradation through the proteasome [81], thus critically modulating the cellular response to AHR activation. AHR can also regulate gene transcription via diverse epigenetic mechanisms, including the regulation of retrotransposons, micro-RNAs and long non-coding RNAs [82,83,84,85]. Several molecules have been found to directly induce the expression of the well-known AHR target gene *CYP1A1*, suggesting possible activation of the receptor in the absence of direct ligand binding [86]. Indeed, nongenomic effects of AHR have been detected, especially in the context of the induction of inflammatory processes. For example, TCDD has been shown to increase the intracellular concentration of calcium, thereby initiating a cascade of reactions ultimately leading to activation of COX2 and the accumulation of inflammatory mediators, such as prostaglandins [73,87]. Moreover, SUMOylation of AHR has been shown to enhance its stability by inhibiting its degradation by the proteasome [88]. Furthermore, SUMOylation can mask ligand binding sites, enhance AHR binding to co-repressors or co-activators, and induce conformational changes, making SUMOylation a critical modulating mechanism of AHR signaling [89]. 

AHR exerts a plethora of cellular functions. It regulates cell proliferation in a cell-type dependent manner [73] and cell adhesion and migration via reorganization of the cytoskeleton [90]. Its pro- and anti-inflammatory function has been extensively reviewed [55,91,92,93]. There is reciprocal regulation of AHR and NF-κB, the master regulator of many inflammatory processes [94,95]. In mouse macrophages stimulated with lipopolysaccharide, the complex of AHR and STAT1 interacts with NF-κB to prevent its transcriptional activity, thus exerting contra-regulatory effects [96]. Moreover, AHR upregulates *SOCS2* which, in turn, represses NF-κB [97,98]. These data highlight the potential anti-inflammatory role of AHR in various cell types. However, the role of AHR in inflammatory reactions, including in AD, is far from understood. AHR has also been demonstrated to have both pro- and anti-oxidative properties [99]. AHR activates NADPH-oxidase and, in turn, induces the production of reactive oxygen species (ROS). In mice, AHR upregulates *Nrf2* in response to its direct binding to the *Nrf2* promoter [100], but whether this holds true in humans remains to be established. AHR has also been identified as responsible for the toxic cellular effects of TCDD via pro-oxidant mechanisms [101,102]. Of note, several mechanisms preventing the deleterious effects of chronic AHR activation have been identified, such as the depletion of the reservoir of endogenous AHR ligands via upregulation of *CYP1A1* [103,104] or overexpression of *AHRR* induced by AHR itself (negative feedback loop) [105].

### 2.2. PXR

PXR is a nuclear hormone receptor (NHR) which must heterodimerize with retinoid X receptor (RXR) to exert transcriptional activity. PXR is among the most promiscuous xenobiotic receptors, as its ligands include a broad range of structurally different molecules, but which are rather species specific. For example, rifampicin can activate human but not mouse PXR, whereas pregnenolone 16α-carbonitrile can only activate mouse PXR. Human PXR activators include drugs (e.g., rifampicin), pollutants (e.g., bisphenols, pesticides) and endobiotics (e.g., bile acids, corticosterone) [106,107,108]. Activation of PXR is a ligand-dependent process and involves interaction with multiple coactivators (e.g., SRC-1 and CREB- CBP/p300) or co-repressors (e.g., NCoR/SMRT) [48,109,110,111,112]. In the absence of ligands, PXR can localize to both the cytoplasm and the nucleus, where it interacts with its corepressors [111,112]. After ligand binding, the heterodimer PXR/RXR binds to specific xenobiotic responsive elements to regulate the transcription of genes coding for phase I (i.e., *CYP3A4*, *CYP1A1*, *CYP4F12*, *CYP2B6* and *CYP2C8*) and phase II (i.e., *SULTs* and *UGT1A1*) enzymes, as well as membrane transporters (e.g., *MDR1*, also called *ABCA1*) [109,111,112,113]. PXR might also be able to exert control of its target genes via epigenetic modifications, such as DNA methylation and noncoding RNA [114].

PXR has pleiotropic functions in regulating bile acid, glucose and lipid metabolism, as well as inflammatory processes, cell proliferation, steroid/endocrine homeostasis, and bone metabolism [58,60]. Similar to AHR, PXR and NF-κB exert mutual repression [115]. NF-κB is able to repress PXR by disrupting the DNA binding of the PXR/RXRα complex on PXR-responsive elements or via post-translational modifications, thereby down-regulating *CYP* expression [116,117,118]. Conversely, PXR activation inhibits the activity of NF-κB in mouse and human cells [115]. Moreover, PXR regulates vitamin K and vitamin D metabolism through the transcriptional control of *CYP3A* and *CYP24* [119,120]. PXR has also been shown to either promote (hepatocytes) or inhibit (colon cancer cells, neuroblastoma cells, cervical cancer cells, hepatocarcinoma cells and lymphocytes) cell proliferation. PXR stimulates cell proliferation via effects on the G0/G1 or G1/S phases and by suppressing cell cycle suppressor genes such as p27 and p130. When acting as a cell proliferation repressor, PXR affects the G2/M phase of the cell cycle, when p21 expression is enhanced and pro-proliferation proteins, like CDCs 20 and 25, are suppressed [60]. PXR also controls processes involved in cell death. For example, it appears to promote hepatocyte survival by upregulating the Bcl-xL and Bcl-2 anti-apoptotic proteins [121]. Moreover, PXR may be protective against DNA damage induced by noxious molecules, such as BaP, by up-regulating the expression of *NQO1*, a phase II detoxification enzyme. This would occur via the activation of the phosphatidylinositol 3-kinase/Akt/Nrf2 pathway [122]. However, this effect might be cell type and context-dependent. 

## 3. Xenobiotic Receptors and Atopic Dermatitis

The skin can absorb pollutants, especially lipophilic molecules. For example, PCBs can easily penetrate the skin to the dermis, as can airborne phthalates and, a fortiori, after topical contact [123,124]. Pesticides and PCBs can accumulate in house dust, which thus represents a primary route of skin contact [124]. Skin is fully equipped to metabolize pollutants, as it possesses phase I and phase II enzymes, drug transporters, and upstream receptors; namely, the xenobiotic receptors AHR, PXR and PPARs, and their mates, LXRs [54,125,126].

Many environmental toxicants target AHR, which may be a potential mechanism eliciting AD [127,128]. However, a large number of environmental pollutants bind to and activate PXR as well [113,129,130,131]. Accordingly, our research group found that xenobiotic metabolism is triggered in the skin of AD patients, regardless of *FLG* status [37]. Consistent with this, several key genes induced in AD skin are PXR and AHR target genes [37,109]. Moreover, metabolites released by skin microbes can also trigger AHR [102,132], which might alleviate AD symptoms [133]. However, the pro- versus anti-inflammatory roles of xenobiotic receptors and their mates in AD remain a matter of debate.

### 3.1. AHR and Atopic Dermatitis

AD skin is characterized by an impaired barrier function, inflammation and dysbiosis. AHR is expressed in a variety of skin cells, including KCs, LCs, T cells, melanocytes, fibroblasts, mast cells and sebocytes [102]. AHR activation has been contrastingly shown to promote [127] or alleviate [134,135] AD. Thus, the role of AHR in AD pathogenesis remains unclear. The pro- versus anti-inflammatory effects of AHR in AD may, in fact, be context-, species-, and ligand-dependent [136]. Some have speculated that activation of AHR signaling might be detrimental in normal uninflamed skin, whereas it might be beneficial in inflamed skin [137]. However, the situation is likely more complex than this and the role of AHR in AD remains to be clarified. Here, we examined the literature in detail to identify conditions or factors favoring one or the other role of AHR in AD. 

#### 3.1.1. When AHR Aggravates or Provokes Atopic Dermatitis

##### Insight from Genetic Analyses

Associations between genetic polymorphisms in AHR and AD are only beginning to be investigated. In a recent study, two AHR single-nucleotide polymorphisms (SNPs) (rs10249788 and rs2066853) were not associated with a higher risk of AD. However, AD patients carrying these SNPs exhibited a significantly higher risk of severe dry skin and allergic rhinitis [138], suggesting they might contribute to abnormal barrier function and have a potential role in the atopic march. rs2066853 causes an arginine to lysine substitution in the acidic sub-domain of transactivation domain of AHR at position 554 (R554K), that might modify secondary structure and reduce AHR stability, as determined with an in silico approach [139]. Another study based on the SALIA cohort investigated the link between traffic-related air pollution and AD in the elderly. The authors found a significant association between all parameters of traffic-related air pollution at the baseline visit and AD incidence, with a higher risk for carriers of the minor allele rs2066853 (e.g., NOx: OR = 3.75, *p* = 0.030 versus OR = 1.34, *p* = 0.317 in non-carriers) [140]. However, the effect of these SNPs on AHR function has not yet been investigated.

##### AD-Related Cellular Abnormalities Triggered by AHR Activation 

The mRNA levels of *AHR*, *ARNT* and *CYP1A1* were increased in the skin of AD patients [127,141,142], suggesting a role of increased AHR signaling in AD. TCDD has been shown to increase the amounts of epidermal CYP1A1 protein [143], as well as *CYP1B1* mRNA in KCs, similar to coal tar, a mixture of > 10,000 substances, including AHR ligands [134]. Moreover, TCDD and coal tar were able to promote hyperkeratosis, a disorder of the stratum corneum observed in AD, in an AHR-dependent manner in 3D organotypic cultures [134,143]. In line with these observations, treatment of human KCs with TCDD or PCB153 triggered the release of IL-8 and IL-6 [142], demonstrating activation of KCs. Furthermore, transgenic mice expressing a constitutively active form of AHR under the control of the *KRT14* promoter (a gene expressed exclusively in basal KCs) developed AD-like symptoms, including itches, epidermal hyperplasia and enhanced dermal inflammatory infiltrate, similar to mice topically treated for 4 weeks with DMBA and FICZ, two AHR ligands [127,144]. The expression of genes involved in cellular detoxification (e.g., *CYP1A1*, *CYP1B1*, *NQO1* and *GSTs*) was increased in the skin of those mice, as was the expression of genes belonging to inflammatory pathways (*TSLP*, *IL13*, *IL18*, *IL1B*, *CXCL5*, *CXCL1*, *IL4R*) and abnormal KC differentiation (*KRT16*) [127,144]. Moreover, these mice exhibited increased levels of serum IgE, IL-4 and IL-5, demonstrating a pro-atopic role of chronic epidermal AHR activation beyond skin [144]. Alloknesis, a sensory abnormality involving hypersensitivity and pruritic paresthesia, was observed in AHR transgenic mice, consistent with observations made in AD patients [127]. This effect seems to be mediated via the upregulation of artemin (*ARTN*) by AHR [127]. Epicutaneous application of diesel exhaust particles (DEPs), the largest source of traffic-related air pollution, or DMBA, the main constituent of the PAHs that make up DEPs, resulted in the upregulation of *ARTN* mRNA levels in the skin, in contrast to topical application with FICZ, a tryptophan-derived endogenous AHR ligand [127], thereby demonstrating a ligand-specific effect. Thus, activation of AHR in the skin by noxious molecules might promote AD via the dysregulation of KC differentiation and the release of inflammatory and neurotrophic mediators by KCs in humans and mice. Nevertheless, the nature of AHR ligands (i.e., environmental pollutants versus endogenous molecules) might be primary in determining the pathogenic outcome of AHR activation in the skin.

##### AHR Activation Promotes Immune Abnormalities Observed in AD

LCs are key immune cells involved in the early development of AD by initiating a Th2 immune response in the epidermis in response to the alarmin TSLP or after activation by allergens or microbe-derived antigens [39,40,41,145]. AHR promotes the expression of *TSLP* by direct binding to its promoter region [127], potentially linking AHR to LC activation which might ultimately lead to AD initiation. In line with this, exposure of mouse skin to BaP increased LC migration and induced a Th2/Th17 immune response mainly via AHR activation [141]. In addition, KCs from AHR-deficient mice produce less GM-CSF, which impairs LC maturation and decreases the Th2-driven contact-hypersensitivity reaction [146]. All together, these results highlight the capacity of AHR activation to induce LC maturation and Th2 inflammation in the skin. However, AHR is expressed in LCs [146,147] and its effects on LCs might be mediated by more than just the release of TSLP or GM-CSF by surrounding KCs. Indeed, in LCs, AHR promoted the activation and metabolism of PAHs, such as DMBA, into active molecules secreted into the epidermal microenvironment and taken up by KCs, where they induced DNA damage [54,147]. This damage to DNA might contribute to abnormal epidermal barrier function and inflammation by increasing the activation of NF-κB and COX2 and by promoting the production of IL-6 and IL-1α in KCs [148,149,150], cellular abnormalities observed in the epidermis of patients with AD. Moreover, PAHs have been identified as one of the main drivers of particle matter-induced inflammation [151]. PAHs are lipophilic molecules with high affinity for AHR. They can easily cross the stratum corneum to reach the living epidermal layers, where they might initiate a Th2-skewed inflammatory response. This might happen via the release of proinflammatory mediators by KCs and activation of LCs, and via additional immune abnormalities contributing to AD onset, flare or symptom exacerbation [127]. Furthermore, several studies using *AHR*-silencing technology and various AHR ligands have demonstrated the preponderant role of AHR in the development of IL-22-producing T cells in several diseases, including AD [152,153], which, in turn, sustains abnormal epidermal barrier function [154]. AHR might also be involved in the development Th17 inflammation; however, this remains controversial and needs to be clarified [136,152,153,155]. 

Thus, increased AHR signaling after topical exposure to AHR high-affinity ligands, such as PAHs, in both KCs and LCs might initiate a Th2/Th17/Th22 immune response and, in turn, significantly contribute to AD development.

##### Role of AHR as an AD Promoter via Oxidative Stress

Oxidative stress can trigger inflammation, and skin and urine from AD patients display increased markers of oxidative stress [37,156,157,158,159]. AHR ligands can be designated as oxidative and anti-oxidative ligands [99]. AHR increases the transcription of genes encoding *CYPs*, whose activities produce ROS [160,161]. Thus, uncontrolled up-regulation of *CYPs* in cells, including in KCs, might generate oxidative stress [57,99]. BaP activates AHR which, in turn, upregulates *CYP1A1* and generates ROS, leading to DNA damage, as evidenced by the production of 8-hydroxydeoxyguanosine and IL-8 in KCs [162,163,164]. Interestingly, IL-8 content in the stratum corneum has been shown to be an indicator of the severity of inflammation in AD lesions [165]. In addition, damaged KCs produce kynurenine, a potent AHR ligand [166]. This metabolite might exert pro-inflammatory effects in skin and be involved in atopic diseases, including AD [167,168]. Moreover, TCDD triggers ROS production in KCs by altering mitochondrial function and dampening the glutathione system [169]. However, other ligands (e.g., ketoconazole and *Bidens pilosa* extract) can bind to AHR without generating ROS [163,170]. Ketoconazole, similar to *Bidens pilosa* extract, was able to activate AHR, as demonstrated by the upregulation of *CYP1A1*, but also, simultaneously, to induce the translocation of Nrf2 to the nucleus and the expression of *NQO1* [163,170], thus producing concurrently a pro-oxidative poison and its antidote. 

Therefore, AHR-induced oxidative stress is likely to be evoked in a ligand-dependent manner and associated with ligand capacity to induce or not a concomitant anti-oxidative response, hence preserving or not the cellular redox balance [171]. However, it remains largely unknown how AHR activators differentially affect the oxidative stress response in the skin and how these processes might affect AD. 

Thus, functional AHR and its downstream target, namely CYP1A1, have been shown to be overexpressed in AD skin [127,141,142]. Moreover, the activation of AHR by noxious molecules, such as pollutants, might sustain or elicit abnormal epidermal barrier function, Th2/Th17/Th22 inflammation, and pruritus in skin via mechanisms which include oxidative stress in KCs (Table 1). In addition, AHR promotes AD development in mice upon constitutive activation that does not require ligand binding [127]. This finding is important because it revealed the skin’s response to chronic AHR activation, regardless of the type of ligand, and the capacity of sustained AHR activation via repetitive exposure to pollutants, SNPs or epigenetic modifications to contribute to AD pathogenesis. 

#### 3.1.2. When AHR Ameliorates AD

##### Promotion of Keratinocyte Late Differentiation and Ceramide Synthesis by AHR

AHR has been shown previously to modulate epidermal barrier function, as illustrated by the significant acceleration in epidermal barrier formation in mouse fetuses after exposure to TCDD [172]. TCDD induced growth arrest in KCs and reduced early KC differentiation, whereas it augmented their late differentiation and the expression of *IL1B*, a gene encoding a cytokine released by KCs to restore epidermal barrier function [169,172,173,174,175], thus pointing to a role of AHR in KC differentiation [175,176]. This was confirmed by work carried out in AHR-deficient mice or KCs treated with AHR antagonists, or showing upregulation of AHR and its downstream gene *CYP1A1* upon KC differentiation [176]. Moreover, AHR ligands were capable of upregulating *FLG* mRNA levels and increasing FLG amounts, regardless of their nature [175]. This might be beneficial in adult AD by contributing to the restoration of natural moisturizing factor (NMF), and thus proper skin hydration [177]. Nevertheless, oxidative stress seems to be required to mediate *FLG* upregulation via AHR because antioxidants were able to block the positive effect of TCDD on the expression of cornified envelope proteins [169]. This is in line with previous work showing the role of oxidative stress in KC differentiation [178,179,180,181,182]. Moreover, the human *FLG* promoter contains an AHR responsive element, also putatively, the *HRNR*, *FLG2* and *LCE3E* promoters [172]. Altogether, those data show that AHR upregulates KC differentiation via direct DNA binding and via indirect pathways requiring mild oxidative stress. As an illustration, coal tar is able to attenuate the deleterious effects of the addition of Th2 cytokines to 3D organotypic cultures generated with KCs isolated from AD patient skin by re-inducing the expression of *FLG*, *LOR* and *HNRN*, and dampening that of *CCL26* [134]. These effects occurr potentially via downregulation of phospho-STAT6 and the translocation of Nrf2 to the KC nucleus, leading to the subsequent upregulation of *NQO1* [134]. However, TCDD was not able to recapitulate all these effects, suggesting there are other players besides AHR involved in bringing the beneficial effects of coal tar to KC differentiation. Nevertheless, the AHR agonist tapinarof (GSK2894512), a bacterial metabolite, was able to ameliorate AD [183,184] via the AHR-Nrf2 axis [185] and upregulation of the expression of late differentiation markers in KCs, including *FLG*, *IVL* and *HNRN* via AHR activation [185], very similarly to coal tar [134,186,187]. 

In AD, the total amounts of ceramides, as well as their composition, are altered (see above). TCDD significantly increased ceramide *de novo* synthesis (i.e., CER 1–7 and CER9 [169]), suggesting another possible beneficial effect of AHR on the epidermal barrier. However, the role of AHR in lipid metabolism and especially in ceramide synthesis deserves further investigation.

Thus, AHR activation might contribute to the restoration of epidermal barrier function in AD (1) via effects on late differentiation markers, (2) via pathways that do not always implicate changes in gene expression (i.e., oxidative stress) and (3) via the synthesis of various ceramides. Interestingly, effects on KC differentiation seem little dependent on the nature of AHR ligands, in contrast to all other effects aforementioned. 

##### Anti-Inflammatory Effects of AHR Activation

The anti-inflammatory effects of AHR goes beyond its role in KCs. AHR is expressed in immune cells, where it can modulate the immune response. In AD, the percentages of circulating regulatory T cells (T_regs_) are consistently found to be increased, but their numbers may be insufficient to counteract ongoing inflammation, or they may even contribute to inflammation by re-differentiating into Th-like cells (e.g., “Th2-like” and “Th17-like” cells) [188,189,190,191,192]. AHR activation by synthetic (e.g., TCDD) or endogenous (e.g., ITE) ligands increased the differentiation of CD4+ T cells into functional T_regs_ exhibiting suppressive activities in mice through various mechanisms, including effects on dendritic cells [55,155,193,194,195,196,197]. In line with this, AHR can modulate the function of Tr1 cells [55]. However, it is not clear yet whether specific subpopulations of FoxP3+ T_regs_ express higher levels of AHR [198], rendering them more susceptible to modulation by AHR agonists. In regard to T_regs_, activation of AHR via FICZ in lymphocytes has given conflicting results so far [155,196,199].

AHR has been reported to the control Th17 immune response. Tapinarof reduced *IL17A* mRNA levels in lymphocytes in an AHR-dependent manner [185], as reported for TCDD [153]. However, AHR activation by FICZ gave discrepant results [153,155,196,199]. 

Patients with AD can experience disease relief in summer owing to immunosuppressive effects of sun. Sun light and UVB induced CYP1A1 in KCs via the production of trace amounts of FICZ and the subsequent activation of AHR [200,201,202]. Thus, one can speculate that FICZ-mediated AHR activation following UV exposure may contribute to localized, beneficial immunosuppression in AD patients via effects on KCs and dendritic cells rather than via direct effects on T_reg_ and Th cell fate.

##### Beneficial Interaction between AHR and the Skin Microbiota

Recently, Ellen von der Bogaard’s team has shown that topical application of coal tar onto the skin of AD patients reduced *Staphylococcus* abundance via upregulation of antimicrobial peptides in a AHR-dependent manner [203], hence highlighting a new role of AHR in the epidermal antimicrobial barrier. Moreover, AHR activation by indole-3-aldehyde, an indole derivative of tryptophan catabolism by the gut microbiota but also found at the skin surface [133], has been shown to alleviate AD-like symptoms in a mouse model of AD induced by MC903. The development of AD symptoms in this mouse model is mediated by the initial increase of TSLP production in KCs which primes LCs to initiate Th2-predominant skin inflammation [40,204]. Indole-3-aldehyde was found to inhibit the MC903-induced expression of *TSLP* in KCs, in vivo and in vitro, via AHR binding to the *TSLP* promoter [133]. Thus, AHR binding to the *TSLP* promoter can alternatively promote [127] or reduce [133] *TSLP* expression, highlighting the dual role of AHR in inflammatory skin reactions and emphasizing the importance of the context, the nature of the ligands, and ligand affinity. 

Recent work showed a dampened tryptophan degradation pathway in the skin microbiota of AD patients when compared to healthy controls [205]. These results suggest lower amounts of AHR anti-inflammatory ligands in AD skin and the putative requirement of chronic activation of AHR by such ligands in the maintenance of healthy skin. In agreement, others found lower levels of indole-3-aldehyde at the skin surface of patients with AD, regardless of the presence of skin lesions, in contrast to the levels of tryptophan, kynurenine and 5-hydroxy-L-tryptophan, all of which remained unchanged [133]. Of note, IL-22 production by Th22 cells in the skin after AHR activation might be protective against microbial infection [206,207,208]. However, recent work has shown little effect of IL-22 in AD pathogenesis [209].

Thus, taken together, these data suggest that the composition of low-affinity AHR ligands produced by the skin microbiota [210] in AD is skewed toward a pro-inflammatory profile due to depletion of indole-3-aldehyde. However, indole-3-aldehyde is synthesized by *Lactobacillus*, whose amounts are not modified in AD skin [205,211,212]. Thus, further work is required to better delineate the role of the skin microbiota in providing the skin with anti-inflammatory AHR ligands.

Thus, AHR activation by ligands, regardless of their receptor affinity and toxicological properties, triggers KC late differentiation, and FLG especially. This might contribute to the restoration of the proliferation/differentiation balance in AD epidermis and the amelioration of skin hydration via the increased production of urocanic acid (UCA) and pyrrolidone carboxylic acid (PCA), components of NMF, which could help ameliorate the epidermal barrier function. Surprisingly, the beneficial effects of ligands on KC differentiation seem totally independent of their capacity to exert proinflammatory effects. Instead, the positive effects might be related, at least in part, to the capacity of the ligands to induce controlled oxidative stress. In contrast, low-affinity AHR ligands, including microbe-derived metabolites, might exert beneficial effects in AD via anti-inflammatory effects (Table 1). As a potential mechanism, high-affinity ligands might induce long activation of the receptor, resulting in damaging effects, whereas low-affinity ligands might induce short activation leading to beneficial effects. This is corroborated by data obtained in AHR transgenic mice [127]. Thus, development of therapeutic treatments aimed at ameliorating AD might focus on the development of low-affinity AHR ligands that combine all the positive effects of AHR activation.

### 3.2. PXR in Atopic Dermatitis

The role of PXR in the skin has been little investigated [213] even though the skin is an important xenobiotic metabolizing organ which expresses several PXR target genes [54,125,213,214,215]. Natural PXR ligands in the skin are not known, but could include progesterone and cholesterol, and their derivatives, bile acids, pregnanes and corticosterone [216,217,218,219,220,221]. The skin is in daily contact with various noxious molecules (e.g., pesticides) contained in water, skin care products and the air, which are potential PXR ligands [108]. Increased concentrations of air pollutants containing PXR ligands are positively associated with AD, and the levels of endocrine disruptors, such as phthalates, are elevated in the dust collected from the bedrooms of children with AD [128,222]. Air pollutants, of which several are lipophilic, can penetrate the skin [123] to activate PXR. However, PXR activation has also been shown to exert anti-inflammatory effects, thus necessitating a closer examination of the role of PXR in the skin and in AD.

#### 3.2.1. Circumstances in Which PXR Aggravates or Provokes AD

##### Constitutive PXR Activation Impairs the Function of the Epidermal Barrier

Skin contact with phthalates, pesticides, bisphenol A and PAHs leads to oxidative stress in KCs, which can evolve into skin disease [129]. Moreover, basal KCs are a major target of topically applied chemicals [223], and many of these molecules activate PXR [224,225,226,227]. Thus, PXR activation in the skin might ultimately lead to skin diseases. To investigate the effects of chronic PXR activation in the skin without being dependent on ligand properties, we generated transgenic mice expressing a constitutively activated human PXR under the control of the *KRT14* promoter. We reported that transgenic mice displayed increased transepidermal water loss (TEWL) and elevated skin pH, abnormal stratum corneum lipids, focal epidermal hyperplasia, activated KCs expressing more *TSLP*, a Th2/Th17 skin immune response and increased serum IgE, thus nicely recapitulating the main features of AD. Furthermore, the cutaneous barrier dysfunction in these mice preceded development of skin inflammation, thereby mirroring the time course of AD development in humans [2,3]. Moreover, further experiments suggested increased PXR signaling in the skin of patients with AD as compared with healthy skin. Indeed, we observed increased nuclear localization of PXR in KCs of AD skin, suggesting constitutive activation of PXR. In line with this, we found a dramatic increase of CYP3A4 immunostaining in the epidermis of AD patients when compared to that of healthy donors [126]. Likewise, the expression of several PXR target genes was significantly enhanced in the skin of AD patients when compared to both healthy subjects and patients with ichthyosis vulgaris (IV) [37]. IV is a monogenetic skin disease resulting from loss-of-function mutations in *FLG*. Nonlesional AD and IV share several common pathological features, such as dry skin, epidermal hyperkeratosis, and abnormal lamellar bodies and lipid bilayers [33]. However, in contrast to AD, IV skin does not display overt inflammation. Thus, we speculated that increased xenobiotic metabolism might promote the shift from noninflammatory dry skin to inflammatory skin. Whether this owes to increased penetration by noxious molecules into skin that has a compromised epidermal barrier remains to be elucidated. Another possible hypothesis is that the enhanced xenobiotic metabolism in AD skin is a consequence of the inflammation and not its cause. However, upregulation of key genes involved in drug, pollutant or chemical metabolism has never been reported in psoriasis, another common inflammatory skin disease with abnormal epidermal barrier function, thus ruling out this hypothesis [228,229]. Interestingly, PXR overexpression or chronic activation in the skin might promote local inflammation via the upregulation of *CYP24*, which, in turn, will increase the catabolism of the active form of vitamin D [120]. So, persistent PXR activation in KCs by environmental pollutants may compromise epidermal barrier function and favor an immune response resembling AD. 

##### Role of PXR in AD via Control of Langerhans Cells

PXR is expressed in LCs and we have shown that PXR deficiency promotes the migration of LCs to skin, draining lymph nodes after topical application with DMBA via the upregulation of *CCR7*, resulting in reduced damage to KCs [54]. These results suggest that PXR activation might be deleterious to KCs, especially after activation by noxious molecules. Consistent with this, LCs treated with DMBA have been shown to upregulate *CYP1B1* and *Epxh1*, thereby producing active DMBA metabolites and increasing its toxicity to surrounding KCs [147]. Thus, one can speculate that activation of PXR in LCs by noxious molecules might trigger their biological activation via the upregulation of phase I enzymes, such as CYP3A4 and CYP1B1. Then, PXR activation, by blocking LC migration, might lead to accumulation of activated molecules in the epidermis and increase their toxicity, thus promoting the release of pro-inflammatory factors by KCs. This is relevant for AD because increased DNA damage to KCs has been observed in AD, as proven by increased levels of 8-OHdG in the serum and urine of patients [159,230]. 

Thus, activation of PXR by environmental pollutants might contribute to the development of AD symptoms by impairing the epidermal barrier function and promoting inflammation (Table 2). Nevertheless, the mechanism through which PXR triggers AD remains to be elucidated but could include effects on oxidative stress, vitamin D, immunity and lipid metabolism.

#### 3.2.2. Circumstances in which PXR Ameliorates AD

To date, PXR has never been investigated as an anti-inflammatory target to treat AD. However, like AHR, PXR displays both pro- and anti-inflammatory effects. PXR is expressed in immune cells, where its activation exerts anti-inflammatory effects. We have shown that PXR is upregulated in activated T-lymphocytes, especially upon activation with TLR ligands, such as LPS and CpG oligodeoxynucleotides [52]. Activation of PXR with specific ligands reduced the production of IFN-γ by LPS/CpG activated CD4^+^ T cells via the upregulation of SOCS1, a master switch for *IFNG* expression [52]. This is in line with other work showing an anti-inflammatory role of PXR via negative regulation of TLR4, a critical determinant of LPS signaling [60]. Moreover, earlier work showed rifampicin, a potent activator of human PXR, to be a suppressor of both humoral and cellular immunity and a powerful immunosuppressive drug [231,232]. Identically to AHR, reciprocal repression between PXR and NF-κB has been shown, at least in the intestine [115,233]. In the colon, PXR-mediated repression of NF-κB target genes appeared to be a critical mechanism by which PXR activation lessened gut inflammation [233]. 

Thus, pharmacological PXR activation with specific ligands might be beneficial to alleviate symptoms via immunosuppressive effects on lymphocytes in patients with chronic adult AD in which a Th1/IFN-γ predominant immune response is observed (Table 2). Similar to AHR, the nature of PXR ligands (low versus high affinity) and duration of PXR activation (chronic versus sequential) might determine the pro- versus anti-inflammatory effects of PXR activation. Moreover, for PXR, a cell-dependent effect might be predictable (KCs versus immune cells). Yet, further work to better characterize the circumstances under which PXR exerts its multiple effects is required.

### 3.3. PPARs in Atopic Dermatitis

The role of PPARs in the skin has been extensively reviewed [66,67,68,234,235,236]. PPARα is present in suprabasal KCs, where it may participate in differentiation and lipid metabolism [237,238]. However, most results have been generated in mice or human KCs treated with supraphysiological doses of PPARα ligands [239]. Similar statements can be made concerning PPARγ and PPARβ/δ [240,241]. Thus, the physiological role of PPARα in human skin remains to be fully deciphered. Nonetheless, topical application of various PPARα ligands has proven to be efficacious in reducing skin inflammation in AD patients [234,235,242,243], in contrast to PPARγ and PPAR β/δ ligands, which have not shown consistent therapeutic effects [241,244,245]. The beneficial effects of PPARα ligands in AD are mediated via effects on lipid metabolism, normalization of KC hyperproliferation and promotion of late KC differentiation, notably by increasing FLG. All these processes, as well as potential anti-inflammatory effects, contribute to restoring the epidermal barrier [234,235,242,243]. 

*PPARA* mRNA levels are reduced in lesional AD skin when compared to healthy and nonlesional skin [34,246], similar to *PPARG* [34]. Cellular abnormalities in lesional AD skin include production of alarmins, also referred as to DAMPs (TSLP, IL-33, HMGB1 and IL-α), by damaged KCs. The reasons why KCs are damaged in AD is not yet known but might result from a combination of genetic (e.g., *FLG* loss-of-function mutations), epigenetic and environmental (e.g., pathogenic microbiota, pollution) factors. Interestingly, *PPARA* mRNA levels were reduced in KCs after UVB irradiation and topical application of a cream containing 5% WY14,643, a well-known PPARα agonist, alleviated UVB-induced erythema [247]. Indeed, PPARα might be dampened in damaged KCs via activation of TLR signaling (e.g., TLR2 and TLR4) by DAMPs (e.g., HMGB1), and subsequent activation of NF-κB, leading to the release of proinflammatory mediators known to inhibit *PPARA* expression (e.g., IL-1β) [248,249] Moreover, PPARα deficiency has been shown to be proinflammatory by promoting the expression of various inflammatory mediators by KCs and immune cells and impairing T_reg_ expansion [66,67,68,246,250,251]. Thus, local and transient *PPARA* downregulation in lesional AD might result from damage inflected on KCs, but then promote tissue regeneration by favoring KC proliferation and the release of inflammatory mediators involved in epidermal barrier recovery. However, these effects might not cover the entire role of PPARα in the epidermis. Moreover, it is likely that persistent PPARα downregulation in this context might, over the long run, become deleterious for the skin. 

Interestingly, PPARα can be activated by xenobiotics, such as phthalates, tributyltin, PCBs, bisphenols, DDT, perfluorooctanoic acid and perfluorooctanesulfonic acid to induce peroxisome proliferation and exert deleterious effects on cells [47,252,253,254,255,256]. Moreover, several genes involved in xenobiotic metabolism are regulated by PPARα [257,258]. Furthermore, DEHP moderately upregulates *PPARA* [259]. Therefore, PPARα might be involved in the toxic effects caused by environmental pollutants. Notably, several studies have demonstrated the impact of such molecules on the metabolism and function of sex steroids through PPAR signaling [254]. This might be relevant in skin diseases, including AD [260]. However, such noxious molecules are low-affinity PPAR activators and further work is required to delineate in vivo the significance of PPAR altered signaling in response to environmental pollutants, including in the skin [252]. Moreover, similar to AHR and PXR, PPAR ligand properties determine the recruitment of co-activators, thus leading to different metabolic responses. Another level of complexity with PPARs is the ability of their ligands to directly regulate metabolic pathways due to “off-target” mechanisms. PPAR ligands can activate kinases able to phosphorylate PPARs and change their transcriptional activity [252]. Moreover, PPAR ligands can exert anti-inflammatory effects in the skin by directly inhibiting pro-inflammatory enzymes (e.g., myeloperoxidase) [261] and modulators (e.g., iNOS, COX-2 and TNF-α) [262,263]. This last point is unfortunately often neglected.

Thus, PPARα ligands exert beneficial effects in AD not only via activation of PPARα but likely also via direct effects (whose proportional contribution remains to be determined) on pathways that remain to be identified. Indeed, these latter PPAR-independent effects of PPARα ligands are poorly understood. Finally, the role of PPARα as a xenobiotic receptor in the skin remains to be fully elucidated.

## 4. Xenobiotic Receptor Mates in AD: LXRs

Both LXRα and LXRβ are expressed in the skin, where their role has already been reviewed [65,67]. Natural LXR ligands include oxysterols, PUFAs, arachidonic acid and PGF2α, which are present in the skin [65]. LXRs inhibit proliferation and promote differentiation of KCs [264,265]. Furthermore, LXR activation exerts anti-inflammatory and anti-oxidant effects [65,67]. Nevertheless, their beneficial effects on epidermal barrier function and in inflammation have so far been attributed to effects on lipid metabolism [65,266,267,268,269]. By extrapolating the beneficial effects of LXR ligands in the skin, the authors of a recent article concluded that LXR ligands might be promising drugs to treat AD [270], although the role of LXRs in AD has not yet been investigated. However, the role of LXRs in human immune cells has been extensively studied and recently reviewed [267,268]. Of note, we found increased levels of LXRβ but unchanged levels of LXRα in AD skin, suggesting a role of LXRβ but not of LXRα in AD (S. Dubrac, unpublished data).

Topical treatment with pharmacological doses of synthetic LXR ligands (GW3965 and T0901317) ameliorated epidermal hyperplasia and skin inflammation, as well as ultrastructural abnormalities, including lamellar body secretion in a mouse model of AD [245]. This is in contrast with the topical application of the natural LXR ligand, 22(R)-hydroxycholesterol, which lacked any benefit. Authors speculated that this naturally-occurring LXR ligand could be metabolized further into an inactive species or act as a bulk lipid, hence destabilizing extracellular lamellar bilayers [245]. Hubaux et al. employed human epidermal equivalents (HEEs) generated with cells from healthy donors and treated with Th2 cytokines to mimic AD, as well as primary cells isolated from AD skin [271]. Concomitant treatment of HEEs with Th2 cytokine cocktail and the LXR agonist GW3965 prevented cellular and molecular abnormalities induced by the Th2 cytokines [271]. However, the authors showed that addition of Th2 cytokines to HEEs inhibited the expression of several genes that are normally increased (e.g., *LCE3A*, *SPRR2A* and *SPRR2B*) [37,272] in AD. 

In a cohort of patients with mild AD, topical treatment with VTP-38543, a LXR selective agonist, significantly increased the expression of genes related to epidermal barrier differentiation (*LOR* and *FLG*) and to lipid metabolism (*ABCG1* and *SREBF1C*) in a randomized, double-blind, vehicle-controlled trial [270]. Moreover, this compound reduced epidermal hyperplasia and restored the balance between KC proliferation and differentiation, as shown by the reduced levels of *KRT16* mRNA [270]. However, VTP-38543 was ineffective at significantly dampening dermal inflammatory infiltrates and down-regulating the mRNA levels of Th17/Th22-related and innate immunity markers. Thus, while LXR ligands might be able to ameliorate epidermal barrier defects by normalizing KC proliferation and lipid metabolism, they appear to lack efficacy for alleviating inflammation in AD. Thus, therapeutic approaches based on LXR ligands might better suit patients with low to mild AD or be proposed as a treatment to prevent AD flare. However, further studies are required to prove their efficacy and determine which LXR isoform is better to target.

LXRs are not primary xenobiotic receptors, but have shown interactions with AHR and PXR (see below), and to a certain extent, are activated by environmental pollutants. Phthalates, organophosphates and fibrates are able to activate LXRα with affinities similar to that of oxysterols, the natural LXR ligands, and to induce changes in the expression of LXRα target genes [273]. Moreover, peaks of ozone (O3) are potentially associated with exacerbation of AD symptoms [274] and with asthma [275]. In the lungs, O3 interacts with cholesterol to produce O3-derived oxysterols whose pro-inflammatory effects might be at least partly mediated via the formation of lipid–protein adducts with LXR. This might dampen LXR signaling and lead to abnormal lipid metabolism and adverse health effects [276]. Observation of the effects of O3 in lungs might also hold true in the skin [277] and in AD [274,278].

Thus, LXRβ expression is increased in AD, potentially as a counteracting mechanism aimed at restoring the barrier via effects on lipid metabolism. Moreover, drugs targeting LXR might exert beneficial effects on low to mild AD and contribute to the prevention of disease flares by ameliorating the quality of the epidermal barrier. However, further research is required on LXR in the context of AD.

## 5. AHR, PXR, LXR and PPAR Crosstalk

Interactions between AHR, PXR, PPARs and LXRs have been demonstrated in various organs, such as the liver and intestine, but so far, not in the skin. However, one can expect that similar crosstalk occurs in the skin as well. AHR and PXR not only regulate overlapping pathways [279,280,281], they also exert mutual regulation [282,283]. Indeed, activation of PXR by rifampicin resulted in a modest induction of *AHR*, but a marked induction of its target genes *CYP1A1* and *CYP1A2* [282]. PXR and LXR upregulate several overlapping genes, including *CYP3A4* and *CD36* [280,283,284], and LXR can downregulate *PXR* [283]. PXR and PPARγ similarly regulate genes involved in lipogenesis, and PXR activation may upregulate *PPARG* expression [283,284]. Moreover, it has been previously shown that activation of AHR by Sudan III disturbs lipid and glucose metabolism by inhibiting PPARα and γ signaling [285] and that the effects of TCDD on diabetogenesis might be mediated via PPAR antagonism [286], thereby corroborating other work [287]. Thus, AHR activation in the skin might contribute to *PPARA* downregulation and, over time, to epidermal barrier dysfunction and inflammation. Interestingly, PPARα has been reported to possess two DNA binding sites on the *CYP1A1* promoter [288] and to control the expression of several genes involved in xenobiotic metabolism, described as PXR and/or AHR target genes [281], thus confirming crosstalk between PPARα and the two other receptors. Moreover, crosstalk between PPAR and LXR has also been identified. PPAR and LXR coregulate a panel of genes involved in cholesterol efflux [289]. Furthermore, a PPAR responsive element (PPRE) has been found in the human LXRα flanking region [290]. This is in line with data showing diminished binding of LXR/RXR to LXRE upon activation of PPARα and PPARγ [291]. Finally, LXRα can interact with all three PPARs to inhibit peroxisome proliferator signaling [292,293]. Thus, PPARs and LXRs play opposite roles in regulating lipid metabolism. 

**Figure 1:**
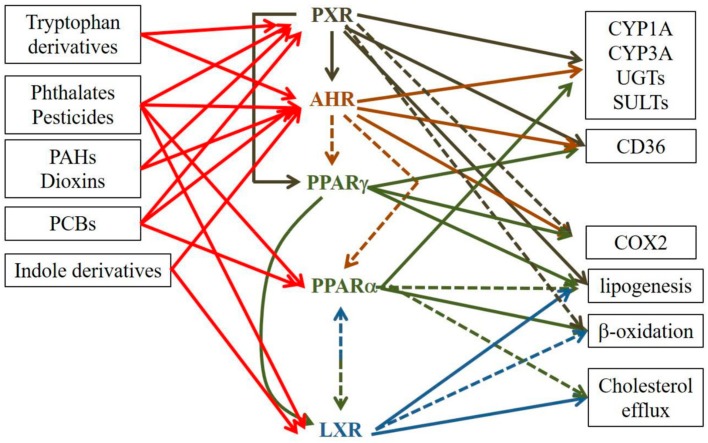
Selected examples of crosstalk between PXR, AHR, PPARs and LXRs. Plain lines designate an activation, whereas dotted lines designate an inhibition.

Therefore, an ongoing challenge is the difficulty of being able to discriminate the effects of one receptor from those of another due to (1) overlapping ligands and (2) the capacity of receptors to interact with each other [294]. Thus, because xenobiotic receptors and their mates engage in crosstalk (Figure 1), the definitive attribution of specific effects to either receptor is fraught with potential error. Therefore, studies of the roles of such receptors in AD should in the future include, as much as possible, experiments to rule out involvement of other xenobiotic receptors. 

## 6. Conclusions

Taken together, there is sufficient evidence to support involvement of xenobiotic receptors and their mates in AD, by either exacerbating or ameliorating the condition, although mechanisms remain to be deciphered, and circumstances under which receptors exert one or the other effect remain to be determined. The current body of literature emphasizes the complexity and often contradictory results and mechanisms associated with receptor activation. The context and the nature of ligands, as well as the duration of the activation, clearly determine the detrimental or beneficial outcome. Indeed, the nature of the ligands—availability, structure, half-life and affinity to the receptor—can lead to diverse cellular responses via processes involving the recruitment of different co-factors [73,252,295]. Noxious molecules, which are, in most cases, high-affinity ligands, might induce prolonged activation of xenobiotic receptors and be deleterious because of ROS production, for example. In contrast, endogenous or microbe-derived ligands, considered for many (not for all) as low-affinity ligands, might only transiently activate the receptors, thus either limiting their potential deleterious effects or even exerting beneficial effects. Therefore, the affinity of ligands to xenobiotic receptors is likely a critical parameter in AD pathogenesis. Other hypotheses include ligand-dependent post-translational modifications of the receptor, thereby affecting its function. Importantly, the effects of ligands have to be distinguished from those owing to receptor activation, because ligands can exert receptor-independent effects [107,252]. Thus, future work should include gene-silencing strategies or receptor antagonists to verify the requirement of the receptor for the observed effects. Discriminating receptor versus non-receptor-mediated effects will serve the development of therapeutically relevant compounds. Moreover, crosstalk between xenobiotic receptors and their mates complicates the attribution of effects solely to one receptor, necessitating complementary experiments to rule out effects from related transcription factors. For example, the use of transgenic mice overexpressing a constitutively activated receptor, and thereby circumventing many of the aforementioned problems linked to ligand or receptor specificity [126,127], demonstrated pathogenic roles of AHR and PXR in AD. However, these observations in mice need to be validated in humans. Thus, understanding the role of xenobiotic receptors and their mates in AD is crucial for limiting exposure to drugs or environmental pollutants that help trigger the disease, and in aiding the development of novel therapeutic approaches able to treat specific disease features (dry skin, epidermal barrier weakness, specific Th inflammation or superinfection) in a personalized medicine approach.

## Figures and Tables

**Table 1:** Dual effects of AHR in atopic dermatitis.

		Pro-AD	Anti-AD
Ligands	High affinity	TCDD, PCB53, PAHs (DMBA, BaP), FICZ	TCDD, ITE
	Low affinity	none	Tapiranof, indole-3-aldehyde
Epidermis		Hyperplasia Hyperkeratosis ↑KRT16 Dry skin (SNPs)	↑late differentiation ↑FLG, ↑IVL, ↑LOR, ↑HNRN, ↑LCE3, ↑FLG2, ↑IL1B ↑CER1-7, ↑CER9
Inflammation		↑IL8, ↑IL6, ↑TSLP, ↑IL1B, ↑CXCL5, ↑CXCL1, ↑IL4RA, ↑GMCSF, ↑IL18 ↑COX2 ↑Th22 ↑Th2 ↑Th17 (?) ↑LC activation and migration	↓CCL26 ↓TSLP ↓Th17 (?) ↑immunosuppressive Tregs
Oxidative stress		↑CYP1A1, ↑CYP1B1 ↑NADPH oxidase ↓glutathione system Mitochondrial dysfunction	↑NQO1 ↑Nrf2
Cell damage		DNA damage ↑NF-κB ↑COX2 ↑IL-6, ↑IL-1α	
Nervous system		Alloknesis ↑ARTN (not FICZ)	
Atopy (atopic march)		↑serum IL-4 & IL-5, ↑serum IgE allergic rhinitis (SNPs)	

↑: upregulation, ↓: downregulation, ?: controversial.

**Table 2:** Dual effects of PXR in atopic dermatitis.

		Pro-AD	Anti-AD
Ligands	High affinity	TCDD, PAHs (DMBA, BaP) Pollutants (??)	Rifampicin, others??
	Low affinity	??	??
Epidermis		Focal hyperplasia Mild hyperkeratosis ↑KRT16 ↑TEWL ↑surface skin pH ↑ki67+ KCs ↑ short chain ceramides	↑FLG
Inflammation		↑IL6, ↑TSLP, ↑IL1B, ↑IL13, ↑CCL27, ↑IL18	↓IFN-γ ↓COX2
		↑Th2	
		↑Th17 (↑IL17A)	
		↑ LC activation	
		↑ILC2	
		dermal inflammatory infiltrate (eosinophils, T cells)	
Oxidative stress		↑CYPs	
Cell damage		↑DNA damage	
Atopy (atopic march)		↑serum IgE, ↑serum IgG1 ↑Th2 in lymph nodes	

↑: upregulation, ↓: downregulation, ??: unknown.

## Abbreviations

ABC	ATP-binding cassette
AD	atopic dermatitis
AHR	aryl hydrocarbon receptor
AHRR	AHR repressor
ARNT	aryl hydrocarbon receptor nuclear translocator
ARTN	artemin
BaP	benzo(a)pyrene
Bcl-2	B-cell lymphoma 2
Bcl-xL	B-cell lymphoma-extra large
CAR	constitutive androstane receptor
CBP	CREB-binding protein
CCR7	C-C chemokine receptor 7
CDC	cell division cycle protein homolog
CER	ceramide
CREB	cAMP response element-binding protein
COX2	cyclooxygenase 2
CYP	cytochrome P450
DAMP	damage-associated molecular pattern
DDT	dichlorodiphenyltrichloroethane
DEP	diesel exhaust particle
DEPH	di-2-ethylhexyl phthalate
DMBA	7,12-dimethylbenz[a]anthracene
FA	fatty acid
FICZ	6-formylindolo[3,2-b] carbazole
FLG	filaggrin
FXR	farnesyl X receptor
GM-CSF	granulocyte-macrophage colony-stimulating factor
GST	glutathione S-transferase
HEE	human epidermal equivalent
HMGB1	high mobility group box 1
HNRN	hornerin
Hsp90	heat shock protein 90
8-OHdG	8-hydroxydesoxyguanosine
IFN	interferon
IL	interleukin
ITE	2-(1′H-indole-3′-carbonyl)-thiazole-4-carboxylic acid methyl ester
iNOS	inducible nitric oxide synthase
IV	ichthyosis vulgaris
IVL	involucrin
KC	keratinocyte
KRT	keratin
LC	Langerhans cell
LCE	late cornified envelop
LPS	lipopolysaccharide
LOR	loricrin
LXR	liver X receptor
MDR	multidrug resistance protein
MRP	multi resistance-related protein
NADPH	nicotinamide adenine dinucleotide phosphate
NCoR/SMRT	nuclear receptor co-repressor
NF-B	nuclear factor kappa-light-chain-enhancer of activated B-cells
NHR	nuclear hormone receptor
NMF	natural moisturizing factor
NOx	nitrogen oxides
NQO1 NADPH	quinone oxidoreductase 1
NRF2	nuclear factor (erythroid-derived 2)-like 2
O3	ozone
OR	odds ratio
PAH	polycyclic aromatic hydrocarbon
PCB	polychlorinated biphenyl
PG	prostaglandin
PPAR	peroxisome proliferator-activated receptor
PUFA	polyunsaturated fatty acid
PXR	pregnane X receptor
ROS	reactive oxygen species
RXR	retinoid X receptor
SNP	single-nucleotide polymorphism
STAT	signal transducer and activator of transcription
SOCS	suppressor of cytokine signaling
SREB1c	sterol regulatory element binding protein 1c
SRC-1	steroid receptor coactivator-1
SULT	sulfotransferase
SUMO	small ubiquitin-like modifier
TCDD	2,3,7,8-tetrachlorodibenzo-p-dioxin
TEWL	transepidermal water loss
TLR	toll-like receptor
TNF-	tumor necrosis factor
Treg	regulatory T cell
TSLP	thymic stromal lymphopoietin
UGT	uridine 5′-diphospho-glucuronosyltransferase
XAP-2	HBV X-associated protein 2
